# Interoception predicts empathy, social anxiety and quality of life in Huntington’s disease

**DOI:** 10.1007/s00415-026-14248-4

**Published:** 2026-11-12

**Authors:** Clare M. Eddy, Hugh Rickards

**Affiliations:** College of Medicine and Health, University of Birmingham and BSMHFT National Centre for Mental Health, Birmingham, UK

**Keywords:** Huntington’s disease, Social cognition, Social anxiety, Quality of life, Interoception, Carers

## Abstract

Previous studies have documented impairments in social cognition and emotional reactivity in Huntington’s disease (HD), with a few highlighting the potential impact on daily functioning and quality of life (QoL). The aims of this study were to explore how patients’ self-reported social cognition was related to interoception, behavioural difficulties, carer burden and the QoL of both HD gene-carriers and their close others. A total of 36 patient-close-other dyads completed a range of scales to assess QoL, self-reported social cognition including affective empathy and perspective taking, social anxiety, interoception, patient apathy and functional capacity, and carer burden. Between group comparisons showed that gene-carriers exhibited lower QoL, emotional awareness, emotion contagion and perspective taking, as well as higher social anxiety and avoidance compared to close others. Patients’ QoL scores were predicted by specific aspects of interoception and affective empathy, but most consistently by social anxiety. Interoceptive attention regulation specifically, predicted patients’ functional capacity. While patient measures did not predict close-other QoL, carer burden was predicted by patients’ apathy and interoception in terms of bodily listening, attention regulation and emotional awareness. These findings suggest that HD is associated with interoceptive dysfunction, which contributes to impairments in emotional awareness and affective empathy, as well as carer burden, and behavioural difficulties as perceived by both patients themselves and their close others. While both impaired interoception and social anxiety directly impact QoL in HD, interoceptive dysfunction may exert a range of indirect influences through various affective symptoms from personal distress to apathy.

**Supplementary Information:**

The online version contains supplementary material available at https://doi.org/10.1007/s00415-026-14248-4.

## Highlights

- HD is associated with interoceptive dysfunction, impacting affective processes.
- Related symptoms range from increased personal distress and social anxiety to apathy and reduced empathic concern.
- Interoception, personal distress and social anxiety influence patients’ QoL.
- Interoception also predicts patients’ functional capacity and carer burden.

**Supplementary Information:**

The online version contains supplementary material available at https://doi.org/10.1007/s00415-026-14248-4.

## Introduction

The inherited neurodegenerative movement disorder Huntington’s disease (HD) features a range of cognitive and behavioural symptoms. Previous studies have shown that cognitive and emotional impairments can impact patients’ quality of life (QoL), i.e. the individual’s perception of their position in life, within the context of the culture and value systems in which they are living, and in relation to their personal goals, expectations, standards and concerns [1]. While physical disability can impair patients’ QoL e.g. [2], mood disorder [3–5] and executive impairments e.g. [6] are also influential. In our previous study [7] we explored the relationship between social cognition and QoL in HD, highlighting the potential impact of patients’ difficulties in reasoning about mental states such as intentions, on QoL within the social and emotional domains. HD gene carriers’ impairments in social cognition align with clinical observation [8] and patient and carer reports [9, 10] of interactions that seem to imply a lack of empathy, which may include socially inappropriate or antisocial behaviour [11–13] and likely underpin early ‘personality changes’ and a range of interpersonal problems [15, 16]. The aims of the current study were to extend our understanding of how HD gene-carriers’ QoL and that of their close others is related to patients’ self-reported social cognition on measures associated with affective empathy (emotion contagion, empathic concern, personal distress) and perspective taking (mind-reading motivation, everyday fantasy and perspective taking tendencies). We also sought to elucidate relationships between social cognition and patients’ behavioural difficulties (apathy, social anxiety and avoidance), interoceptive skills and functional capacity, as well as identifying which factors may predict carer burden.

Behavioural problems, particularly those associated with motivation, such as apathy, are common in HD [16] and track with disease progression. Social withdrawal is seen at varying levels, is associated with psychiatric, cognitive and behavioural issues such as mood swings, and affects caregiver burden [17]. A recent review suggested that social cognitive impairments seen in movement disorders are associated with motivational problems ranging from apathy to impulsivity [18]. More specifically, qualitative research has documented the association between apathy and relationship satisfaction [19], with this latter variable also being likely to exert a crucial impact on QoL. Previous studies have also reported associations between apathy and aspects of cognition such as executive functions and instrumental learning [20], although some studies report correlations with emotion recognition rather than cognitive measures [21].

While apathy has been quite extensively studied in HD, data for social anxiety is lacking, and few if any studies have explored relationships between social cognition and social anxiety and avoidance specifically. Given the paucity of previous research on this topic, we cannot assume that HD itself produces a specific social anxiety syndrome. However, it makes sense to think that social anxiety could be another symptom that affects social motivation that would be experienced by many gene-carriers, given the prevalence of anxiety symptoms more generally [22] and the potential for anxiety in social situations due to the visibility of symptoms and related embarrassment or self-consciousness. Beyond observable movement disorder, symptoms that impair speech and communication also lead to anxiety and impact QoL [23]. Individuals at an early stage or who don’t exhibit obvious cognitive or motor symptoms could still experience increased self-consciousness or self-monitoring for the appearance of signs or symptoms that could suggest progression of HD, and this in itself could contribute to social anxiety. Therefore, in addition to lower QoL and higher apathy, we hypothesised that gene-carriers would exhibit greater social anxiety when compared to close other controls. Furthermore, given the prevalence of apathy in HD, evidence of correlations between performance on measures of social cognition and of apathy [7, 21, 24, 25], and non-clinical research on social anxiety [26], we hypothesised there would be positive correlations between affective empathy and social anxiety but negative correlations between apathy and perspective taking, or more precisely, motivation to try to understand other people’s mental states.

Interoception, which refers to the awareness of internal physiological states, based on sensing and interpreting cardiovascular, gastrointestinal, respiratory and cutaneous signals [27], can be disrupted in a range of neurological and psychiatric conditions [28]. There is evidence that interoception contributes to affective empathy (i.e. in the ability to feel into others’ emotional states). For example, cortical processing of cardiac output has been correlated with empathic concern as measured by the IRI supporting the interpretation that visceral activity may contribute to affective judgments about others [29]. Furthermore, via its role in affective empathy, interoception may influence broader social cognition in terms of perspective taking and in turn, cognitive empathy [30]. Impairments in social cognition including those pertaining to affective empathy deficits have been quite extensively documented in HD [31]. However, there has been surprisingly little investigation of how interoception could contribute to gene-carriers’ social cognitive impairments. Gene-carriers do have difficulties with sensory organisation [32] and show abnormal stimulus salience/pain perception on somatosensory tasks involving thermal and electrical stimuli [33]. It has also been shown that sensory-perceptual processing predicts time to diagnosis in HD [34]. Difficulties with integrating interoceptive signals with affective labels could help to explain why a few studies have identified alexithymia i.e. difficulty understanding and communicating one’s emotions, in HD [24, 35], and why patients have difficulties with inferring emotional states from the same facial expressions that they can accurately label in terms of physical characteristics such as age [36]. We hypothesised that participants with HD would exhibit impairments in interoception alongside reduced affective empathy. In addition, because internal bodily states should influence one’s own motivational states [37], we expected to see correlations between interoceptive skills and any symptoms of apathy and social anxiety. The measure used in the current study explored interoception across domains such as perceiving and regulating attention to bodily signals, as well as related inferences and emotional reactions [28, 38].

Correlations between social cognition and QoL in HD emphasise the role of relationships with close others, and social interaction, in determining wellbeing. Importantly, previous studies have shown that behavioural difficulties associated with HD, such as apathy or frontal-type behaviours, can affect the wellbeing of carers or family members even more than the patients themselves [19, 39, 40], and burden carers more than motor symptoms [41]. Emotional insight and feelings about social relationships are correlated with apathy, irritability and anxiety in HD [42] and partners can feel a fading connection with gene-carriers who seem to withdraw affection [43] or ‘change completely’ such that they don’t feel they know them anymore [44]. While patients themselves can report feeling isolated because of misunderstandings with caregivers, relationship friction contributes to the burden felt by those close others who are trying to balance their provision of practical and emotional support with addressing their own needs and maintaining a healthy relationship with the gene-carrier [43, 45]. Therefore social cognitive deficits and related behavioural problems could influence patients’ functional capacity [46, 47], as well as compromising the emotional wellbeing of HD gene-carriers and their close others.

In summary, the current study aimed to identify any differences between HD gene-carriers versus a close other control group in terms of self-reported social cognition, interoception and QoL, as well as to investigate relationships between these variables, patients’ functional capacity and behavioural difficulties, and carer burden. We hypothesised that patient QoL and functional capacity would be predicted by both self-reported social cognition and their behavioural difficulties, as well as interoception, given our previous hypothesis that interoception would contribute to affective empathy as well as the patients’ awareness of their own emotional states. Furthermore, we hypothesised that patients’ self-reported social cognition, interoception and behavioural difficulties would also predict aspects of close other QoL and carer burden.

## Methods

### Study design and sample

This was a cross-sectional independent groups design study. Participants were recruited as dyads consisting of one HD gene-carrier (the patient) plus one close other who was gene negative. Close others provided some additional information about the patient as well as control data for comparison. All participants were recruited through one of two sources: a specialist clinical service for HD in Birmingham, UK or via the national organisation, the Huntington’s Disease Association. Gene-carriers had received a positive genetic test result for HD at least 6 months prior to enrolment. Exclusion criteria were any previous diagnosis of autistic spectrum disorder, current acute mental health crisis, concurrent involvement in psychological counselling, or disease severity affecting the ability to provide consent or to be able to complete the study tasks. Some gene-carriers were assisted by their close others to complete the questionnaires.

### Testing procedure

The study received NHS and institutional ethical approvals. The vast majority of participants completed questionnaires online after providing written informed consent. A few participants preferred to complete the same questionnaires on paper and the data was added into the online system by a research assistant. Participants received a small gift voucher for participation. The questionnaires were split into two parts and began with the demographic and QoL questionnaires, followed by the other measures. Anonymity was maintained using unique ID codes. Patients completed all questionnaires about themselves, while close others were asked to complete most measures about themselves but also an apathy measure about the gene-carrier.

### Self-report social cognition scales

#### Interpersonal reactivity index (IRI)

This widely used self-report measure [48, 49] is thought to encompass both cognitive (perspective taking; fantasy) and affective (empathic concern; personal distress) aspects of empathy. Participants decide how well each statement describes them. Each statement falls into one of four subscales: Perspective taking (PT) e.g. *“I try to look at everybody’s side of a disagreement before I make a decision”;* personal distress (PD) e.g. “*I sometimes feel helpless when I am in the middle of a very emotional situation”;* fantasy e.g. “*After seeing a play or movie, I have felt as though I were one of the characters”;* and empathic concern (EC) e.g. *“I am often quite touched by things that I see happen”.* Each item is rated from does not describe me well to describes me very well, and scores can range from 0 to 28 for each of the four subscales, which each have 7 items and are best examined separately rather than in combination [50]. Recent research indicated good to excellent test–retest reliability [51] supporting the use of the IRI as a trait measure. The scale has been administered previously in HD e.g. [49].

#### Emotion contagion scale (ECS)

The ECS [52] measures susceptibility to other people’s emotions, and automatic processes likely to contribute to affective empathy. It has high reliability and construct validity, containing 15 items such as “*Being with a happy person picks me up when I’m feeling down*”. Items pertain to happiness, anger, fear, love or sadness. Each item is rated from ‘*never*’ to ‘*always*’, with total score ranging from 15 to 60. A number of studies suggest that it is preferable to apply the ECS as a unidimensional model using total scores rather than emotion or valence specific subscales [53–55]. Few if any studies have used this measure before to investigate HD.

#### Mind reading motivation (MRM)

This scale assesses general tendencies towards thinking about the mental states of others rather than ability. The developers [56] showed it has good test–retest reliability and can be associated with prosocial skills such as teamwork, as well as measuring beyond trait empathy. It contains 13 items such as “*It is pointless to try to see things from other people’s points of view*”, that are rated from strongly disagree to strongly agree to generate one total score (from 13-91). It is unlikely this measure has been used previously in HD.

### Scales assessing behavioural difficulties

#### Liebowitz social anxiety scale

The LSAS includes two subscales with 24 items in each, rated on a Likert scale between 0 and 3. For the anxiety subscale responses are rated from none to severe, and for the avoidance subscale items are rated from none to usually [57]. Suggested clinical cut offs for the scale are: 30 for social anxiety, 60 for generalised social anxiety disorder [58]. It has good reliability and validity including in relation to internal consistency and convergent validity [59, 60] but may not have been used before in HD.

#### Apathy evaluation scale (AES)

This scale [61] has 18 items that can be completed as self-report or by informants or clinicians. Statements relate to an individual’s interests or responses to daily activities and give an indication of initiative, drive or motivation (e.g. “someone has to tell her/him what to do each day”). Statements are rated using a Likert scale from 1 to 4, hence total scores can range from 18 to 72. Scores above 38 are typically considered to reflect clinically significant apathy. Previous studies confirm its reliability and validity, including in terms of assessing apathy as opposed to depression or anhedonia [61, 62]. This scale is recommended for the assessment of apathy severity in HD [63].

### Interoception questionnaire

#### Multidimensional assessment of interoceptive awareness 2 (MAIA-2)

This state-trait questionnaire [38, 64] contains a total of 37 items, and encompasses 8 subscales with each item rated from 0 = never to 5 = always. *Noticing* pertains to the awareness of uncomfortable, comfortable, and neutral body sensations; *Not-Distracting* looks at the tendency not to ignore or distract oneself from sensations of pain or discomfort; *Not-Worrying* focuses on the tendency not to worry or experience emotional distress with sensations of pain or discomfort; *Attention Regulation* assesses the ability to sustain and control attention to body sensations; *Emotional Awareness* concerns awareness of the connection between body sensations and emotional states; *Self-Regulation* queries the ability to regulate distress by attention to body sensations; *Body Listening* considers active listening to the body for insight; *Trusting* provides an indication of experience of one’s body as safe and trustworthy. Reliability and validity are good [38, 64], with a higher score (total from 0 to 185) indicating greater interoceptive sensibility. The scale has been translated into many languages and applied in many clinical populations but there are no published studies involving HD.

### Measures of quality of life and carer burden

#### RAND health questionnaire SF36

This self-report Qol measure [65] contains 8 subscales: physical functioning, bodily pain, social functioning, energy or fatigue, role limitations due to physical health problems, role limitations due to emotional problems, emotional well-being, and general health perceptions. Both patients and close others completed this scale for themselves. Higher scores for each subscale indicate better QoL. Internal consistency and test–retest reliability have been shown to be sufficiently high in HD [63]. Scoring was based on standard RAND recommendations [66].

#### Huntington’s disease quality of life instrument

HD gene-carriers completed the disease-specific HDQoL measure [67, 68] that includes 40 items, and generates scores for 5 subscales: cognitive, physical, mood-self, worry, and services. Items are rated from never to all of the time and scores range from 0 to 100 for each subscale with higher scores reflecting superior QoL. Reliability and internal consistency is high [67, 68].

#### Carer burden inventory (CBI)

The majority of close-others (23/36) also self-identified as carers for their patient. These individuals completed the CBI [69] which was developed with caregivers of those with cognitive impairment and Alzheimer’s disease. It has 24 items and 5 subscales: developmental, emotional, social, time dependence and physical burden, each having 5 items except the latter subscale, with item ratings from 0 = not at all to 4 = very descriptive. Internal consistency is very good [70]. While caregiver burden has been explored in a number of studies in relation to HD, application of the CBI specifically appears rare.

### Assessment of functional capacity

#### Unified Huntington’s disease rating scale: total functional capacity

This short instrument [71] allows for an assessment of everyday functioning in HD related to activities of daily living such as occupation, household and self-care, and indicators of independence. Raw scores can range from 0 to 13.

### Analyses

Analyses were conducted using IBM SPSS version 30.0. Normality tests (Shapiro–Wilk) indicated non-normal distributions for a few variables, and non-parametric statistics were applied where appropriate, including for analyses conducted on group sizes < *n* = 30. Stepwise regressions were used to address the study hypotheses, with specific subscales included based on initial correlational analysis. Total scores were used as DVs but not as IVs where individual subscales were included as predictors.

## Results

### Participant characteristics

The sample consisted of 33 patient-close other dyads plus 3 extra patients and 3 extra close-others. As shown in Table 1, the groups were matched for age and ethnicity with a slight imbalance in terms of gender. This table provides further information including clinical characteristics of the HD gene-carrier group. Data were missing for a few of the study measures for a few participants (2 close others and 1 patient) and only 22 close others identified as being in a carer role. Therefore, while between-group comparisons involved all patients (*n* = 36) v close others (*n* = 36) as controls, correlations were conducted on 33 dyads, apart from those concerning the AES (31 pairs) or CBI (22 pairs).

**Table 1:** Sample characteristics

Group	Variable	Details
Close others	Gender	Female = 15, male = 21
	Ethnicity	Caucasian = 28, black African = 8
	Age	Mean = 50.37, SD = 15.81, median = 52, range = 24–72
	Relationship to gene-carrier*	Partner = 26, sibling = 3, close friend = 2, step sibling = 1, niece = 1, no connection = 3
HD gene-carriers	Gender	Female = 22, male = 14
	Ethnicity	Caucasian = 28, black African = 8
	Age	Mean = 50.11, SD = 15.02, median = 53, range = 24–74
	CAG repeat	Range = 40–63, 28/29 values between 40–49
	TMS^†^	Mean = 28.72, SD = 22.32, median = 23, range = 1–77
	DCL^†^	Range = 0–4: 0 = 3; 2 = 1; 4 = 14
	Medications	None = 14, atypical anti-psychotic + tricyclic/SSRI/SNRI = 5, sertraline = 5, tetrabenzine = 4, olanzapine = 3, mirtazepine = 2, aripiprazole = 1, clonazepam = 1, dalzanemdor = 1

^*^The sample consisted of 33 patient-close other dyads plus 3 extra patients and 3 extra close-others. ^†^Clinical data were available for a subset of gene-carriers (*n* = 18 +), including most patients recruited through the clinical source

### Descriptive statistics

Descriptive statistics for the two groups are shown in Table 2, while Supplementary Table 1 (Online Resource 1) lists the significant relationships identified in correlational analyses. It is notable that approximately 2/3 patients scored within the clinical range for social anxiety according to the LSAS, while this was only the case for up to 1/3 of close others. There were similar findings in terms of apathy, with 87.5% of patients scoring above the AES clinically relevant cut-off as rated by their close others. Table 3 includes the statistical comparisons for hypothesis 1, while Table 4 gives the statistics relevant to hypotheses 2–5.

**Table 2:** Descriptive statistics for Huntington’s disease gene-carrier and close other groups

Measure	Group	Mean	SD	Median	Range	Shapiro–Wilk (normality test) statistic, p-value
UHDRS TFC	PT	9.10	3.27	10	2–13	.903, .008
MRM	PT	53.88	10.84	54.50	28–72	.966, .359
	CO	56.94	9.49	58.00	32–80	.987, .950
LSAS anxiety	PT	37.12	20.53	39.00	0–70	.953, .155
	CO	23.85	15.62	21.00	0–53	.949, .125
LSAS avoidance	PT	36.35	21.86	43.00	0–72	.926, .023
	CO	21.18	16.86	16.00	1–64	.911, .010
ECS total	PT	39.09	8.62	41.00	20–53	.950, .124
	CO	43.76	5.44	44.00	30–54	.977, .685
MAIA-2 total	PT	93.32	24.05	100.50	23–137	.837, <.001
	CO	95.55	21.73	95.00	45–140	.978, .717
MAIA-2 noticing	PT	2.76	1.27	3.13	0–4.50	.892, .003
	CO	2.86	1.32	3.00	0–5	.963, .314
MAIA-2 not distracting	PT	2.13	1.17	2.00	0–5	.967, .391
	CO	1.76	1.02	1.83	0–5	.944, .087
MAIA-2 not worrying	PT	2.44	0.78	2.40	0–4.20	.948, .104
	CO	2.55	0.82	2.80	0.6–4.2	.959, .244
MAIA-2 attention regulation	PT	2.61	1.05	2.86	0–4.29	.926, .024
	CO	2.65	0.98	2.86	0–4.29	.917, .015
MAIA-2 emotional awareness	PT	2.75	1.28	2.90	0–4.80	.947, .098
	CO	3.32	1.25	3.60	0–5	.947, .108
MAIA-2 self-regulation	PT	2.34	1.21	2.50	0–5	.982, .845
	CO	2.59	1.11	2.75	0–5	.976, .650
MAIA-2 listening	PT	2.27	1.45	2.67	0–5	.924, .021
	CO	2.11	1.38	2.33	0–5	.945, .098
MAIA-2 trusting	PT	3.05	1.44	3.00	0–5	.941, .064
	CO	2.98	1.37	3.00	0–5	.960, .252
IRI perspective taking	PT	14.24	6.86	14.00	0–28	.985, .915
	CO	16.88	5.88	16.00	4–28	.961, .270
IRI empathic concern	PT	17.15	6.19	16.00	3–28	.982, .845
	CO	19.15	4.41	19.00	7–26	.955, .190
IRI personal distress	PT	13.09	6.26	13.50	1–28	.981, .824
	CO	12.06	4.85	12.00	2–23	.983, .868
IRI fantasy	PT	11.94	5.54	12.00	1–24	.983, .890
	CO	14.36	4.97	14.00	6–24	.967, .394
SF36 physical functioning	PT	50.56	36.39	50.00	0–100	.905, .008
	CO	86.39	21.20	95.00	20–100	.694, <.001
SF36 role physical	PT	38.19	42.88	25.00	0–100	.757, <.001
	CO	81.94	31.96	100.00	0–100	.559, <.001
SF36 role emotional	PT	40.74	43.72	33.33	0–100	.747, <.001
	CO	81.48	32.31	100.00	0–100	.588, <.001
SF36 energy	PT	46.25	22.82	48.00	0–95	.974, .612
	CO	60.42	26.49	60.00	10–130	.981, .806
SF36 emotional wellbeing	PT	59.11	22.75	58.00	16–100	.961, .286
	CO	67.33	20.92	72.00	4–104	.964, .327
SF36 social functioning	PT	57.98	30.05	50.00	12.5–100	.882, .002
	CO	72.91	23.43	75.00	25–100	.899, .005
SF36 pain	PT	62.78	33.03	62.50	10–100	.793, <.001
	CO	72.92	24.63	77.50	0–100	.904, .007
SF36 general health	PT	44.86	21.23	45.00	5–80	.951, .157
	CO	62.64	23.12	62.50	5–100	.954, .172
AES total	PT	50.28	10.74	49.00	3–71	.968, .458
HDQoL total (%)	PT	52.19	23.46	48.54	0–97.08	.980, .753
HDQoL physical	PT	58.62	27.02	61.67	15–100	.933, .048
HDQoL cognitive	PT	45.25	27.93	45.83	0–100	.908, .010
HDQoL mood	PT	46.91	24.06	38.89	0–97.22	.922, .023
HDQoL worry	PT	44.29	24.53	47.22	0–88.89	.962, .304
HDQoL services	PT	71.91	26.49	75.00	0–100	.893, .004
CBI total	CO	36.17	18.45	38.0	0–63	.954, .349
CBI time	CO	12.04	5.80	13.0	0–20	.944, .216
CBI developmental	CO	10.09	5.95	10.0	0–18	.909, .039
CBI physical	CO	5.87	4.15	5.0	0–15	.953, .335
CBI social	CO	5.52	3.93	5.0	0–12	.941, .188
CBI emotional	CO	2.65	3.97	1.0	0–14	.709, <.001

KEY: *AES* Apathy Evaluation Scale; *CBI* Carer Burden Inventory; *CO* Close other group; *HDQoL* Huntington’s Disease Quality of Life scale; *IRI* Interpersonal Reactivity Index; *LSAS* Liebowitz Social Anxiety Scale; *MAIA-2* Multidimensional Assessment of Interoceptive Awareness 2; *MRM* Mind Reading Motivation scale; *PT* Patient (HD gene-carrier) group; *SD* Standard deviation; *SF36* Rand Health Questionnaire; *UHDRS TFC* Unified Huntington’s Disease Rating Scale Total Functional Capacity

**Table 3:** Significant group differences on study measures

Measure*	Statistics	*P*-value
IRI fantasy	t(65)=−1.883	.032
IRI PT	t(65)=−1.691	.048
ECS	t(65)=−2.658	.005
MAIA-2 Emotional awareness	t(65)=−1.821	.037
LSAS anxiety	(t(65)=2.971	.002
LSAS avoidance	MWU = 336.5	.005
SF36 Physical functioning	MWU = 1034	< .001
SF36 Role physical	MWU = 998.5	< .001
SF36 Role emotional	MWU = 964	< .001
SF36 energy	t(70)=− 2.431	.009
SF36 Social functioning	MWU = 833.5	.034
SF36 General health	t(70)=−3.398	< .001

^*^The remaining measures didn’t reach statistical significance (*p*≤.05): IRI subscales empathic concern and personal distress; MAIA-2 subscales not distracting, noticing, trust, self-regulation, attention regulation, not worrying and listening; SF36 subscales emotional wellbeing and pain

**Table 4:** Significant regression models for Hypotheses 2–5

Hypothesis	Dependent variable	Independent variables	Co-efficients	Model statistics	% variance explained*
2 PT	IRI EC	MAIA-2 attention regulation	*β*=0.436, *t* = 2.744	F(1,32)=7.529, *p*=.010	16.5%
	IRI PD	MAIA-2 not worrying	*β*=−0.575, *t* =− 3.973	F(1,32)=15.787, *p*<.001	30.9%
	LSAS anxiety	MAIA-2 not worrying	*β*=−0.496, *t* =− 3.233	F(1,32)=10.454, *p*=.003	22.3%
	LSAS avoidance	MAIA-2 not worrying	*β* = −0.438, *t* =− 2.753	F(1,32)=7.580, *p*=.010	16.6%
2 CO	IRI PD	MAIA-2 self regulation	*β* =−0.356, *t* = 12.122	F(1, 31)=4.501, *p*=.042	12.7%
	IRI EC	MAIA-2 self regulation	*β*=0.388, *t* = 2.343	F(1,31)=5.491, *p*=.026	12.3%
	ECS	MAIA-2 self regulation	*β*=0.358, *t* = 2.134	F(1,31)=4.552, *p*=.041	10.0%
3	AES	IRI EC	*β*=0.394, *t* = 2.395, *p*=.023	F(2,29)=10.709, *p*<.001	38.5%
		ECS	*β*=0.354, *t* = 2.156, *p*=.040		
	LSAS anxiety	IRI PD	*β*=0.566, *t* = 3.881	F(1,32)=15.063, *p*<.001	29.9%
	LSAS avoidance	IRI PD	*β*=0.402, *t* = 2.486	F(1,32)=6.181, *p*=.018	13.6%
4	UHDRS TFC	MAIA-2 attention regulation	*β*=0.529, *t* = 3.295	F(1,28)=10.855, *p*=.003	25.4%
	SF36 Physical Function	MAIA-2 not worrying	*β*=0.604, *t* = 4.291	F(1,32)=18.410, *p*<.001	34.5%
	SF36 Role physical	IRI PD	*β* = −0.429, *t* =− 2.968, *p*=.006	F(2,31)=9.457, *p*<.001	33.9%
		MAIA-2 listening	*β* = −0.364, *t* =− 2.516, *p*=.017		
	SF36 Role emotional	LSAS anxiety	*β* =−0.571, *t* =− 3.930	F(1,32)=15.447, *p*<.001	30.4%
	SF36 Emo wellbeing	LSAS anxiety	*β* = −0.536, *t* =− 3.916	F(2,31)=13.330, *p*<.001	42.8%
		MAIA-2 listening	*β* = −0.298, *t* =− 2.176, *p*=.037		
	SF36 Energy	LSAS anxiety	*β* = −0.464, *t* =− 2.961, *p*=.006	F(1,32)=8.766, *p*=.006	19.0%
	SF36 Social functioning	MAIA-2 not worrying	*β*=0.510, *t* = 3.350	F(1,32)=11.222, *p*=.002	23.6%
	SF36 Pain	IRI PD	*β*=−0.538, *t* =− 3.609	F(1,32)=13.028, *p*=.001	26.7%
	SF36 General Health	IRI PD	*β*=−0.445, *t* =− 2.814	F(1,32)=7.917, *p*=.008	17.3%
	HDQoL Total	LSAS anxiety	*β*=−0.725, *t* =− 5.960	F(1,32)=35.524, *p*<.001	51.1%
	HDQoL Physical	LSAS anxiety	*β*=−0.642, *t* =− 4.784	F(1,32)=22.887, *p*<.001	39.9%
	HDQoL Cognitive	LSAS anxiety	*β*=−0.642, *t* =− 4.741	F(1,32)=22.482, *p*<.001	39.4%
	HDQoL Mood	LSAS anxiety	*β*=−0.670, *t* =− 5.107	F(1,32)=26.085, *p*<.001	43.2%
	HDQoL Services	LSAS anxiety	*β*=−0.474, *t* =− 3.041, *p*=.005	F(1,32)=9.249, *p*=.005	20.0%
	HDQoL Worry	LSAS anxiety	*β*=−0.553, *t* =− 4.185, *p*<.001	F(2,31)=13.610, *p*<.001	43.3%
		IRI Fantasy	*β*=−0.338, *t* =− 2.558, *p*=.016		
5	CBI Total	AES	Β=−0.496, t =− 3.010, *p*=.007	F(2,20)=8.688 *p*=.002	41.1%
		MAIA-2 not distracting	*β*=0.412, *t* = 2.300, *p*=.021		
	CBI Physical	MAIA-2 emotional awareness	*β*=−0.603,* t* =− 3.384	F(1,21)=11.450, *p*=.003	3.2%
	CBI Social	AES	*β*=−0.578, *t* =− 3.290, *p*=.004	F(2,20)=9.049, *p*=.002	42.3%
		MAIA-2 Listening	*β*=−0.363, *t* =− 2.222, *p*=.038		
	CBI Time	MAIA-2 attention regulation	*β*=−0.472, *t* =− 2.450	F(1,21)=6.005, *p*=.023	18.5%
	CBI Development	MAIA-2 not distracting	*β*=0.476, *t* = 2.479	F(1,21)=6.144, *p*=.022	19.0%

KEY: *AES* Apathy Evaluation Scale; *CBI* Carer Burden Inventory; *CO* Close other group; *HDQoL* Huntington’s Disease Quality of Life scale; *IRI* Interpersonal Reactivity Index; *LSAS* Liebowitz Social Anxiety Scale; *MAIA-2* Multidimensional Assessment of Interoceptive Awareness 2; *PT* Patient (HD gene-carrier) group; *SF36* Rand Health Questionnaire; *UHDRS TFC* Unified Huntington’s Disease Rating Scale Total Functional Capacity. * Percentage shown is based on R^2^ adjusted value

### Hypothesis 1. Patients will have lower scores for self-report social cognition scales, interoception, and QoL, but higher social anxiety and avoidance than close others

In relation to self-reported social cognition, HD gene-carriers exhibited significantly lower scores for fantasy and perspective taking than close others, but there was no difference for mind reading motivation. Patients also exhibited lower emotion contagion, and interoception specifically in terms of emotional awareness. Social anxiety and avoidance were also higher in patients, who demonstrated significantly lower scores on most SF36 subscales: physical, role physical, role emotional, energy, social functioning and general health.

### Hypothesis 2. Interoceptive skills will predict affective empathy, apathy, social anxiety and avoidance

For gene-carriers, the best model for empathic concern contained the predictor interoception: attention regulation, while the best model for personal distress contained the predictor interoception: not worrying. Patients’ emotion contagion scores were not correlated with any of their interoception scores.

For close others, the best model for empathic concern as DV (and also for emotion contagion as DV) contained the predictor interoception: self-regulation. The best model for PD contained the predictor interoception: not worrying, as for patients.

For patients, both social anxiety and social avoidance were predicted by interoception: not worrying. Although interoception: attention regulation was correlated with apathy Supplementary Table 1 it was not a significant predictor in the regression model.

### Hypothesis 3. Patients’ self-reported social cognition will predict apathy and social anxiety

HD gene-carriers’ apathy as rated by their close others was predicted by their self-rated EC and emotion contagion. Patients’ ratings for PD predicted both their self-rated social anxiety and social avoidance.

### Hypothesis 4. Patient QoL and functional capacity will be predicted by apathy, social anxiety and avoidance, self-reported social cognition and interoception

Patients’ functional capacity was predicted by interoception: attention regulation.

In relation to QoL as measured by the SF36, the best model for physical functioning contained the predictor interoception: not worrying, and role physical was predicted by PD and interoception: listening. Role emotional was predicted by just social anxiety, while the best model for emotional wellbeing contained the predictors social anxiety and interoception: listening. The best model for energy contained the predictor social anxiety, and social functioning was predicted by interoception: not worrying. The best model for pain and for general health contained the predictor PD.

The best model for HDQoL total score and for the physical subscale contained the predictor social anxiety. The best model for the cognitive, mood and services subscales also contained the predictor social anxiety. Gene-carriers’ worry was predicted by both social anxiety and fantasy.

### Hypothesis 5. Close other QoL and carer burden will be predicted by patients’ apathy, social anxiety and avoidance, interoception and self-reported social cognition

There were no significant correlations between close other SF36 subscale scores and patient measures of apathy, social anxiety, interoception or self-reported social cognition. There were also no significant correlations between the SF36 subscale scores for close others and any of the subscale scores for patients’ QoL.

The best model for CBI scores included the predictors apathy and interoception: not distracting. The best model for physical burden included the predictor interoception: emotional awareness, and the best model for social burden included the predictors apathy and interoception: listening. Just one predictor (interoception: attention regulation) was in the best model for time burden, and this was also the case for developmental burden (interoception: not distracting). There were no significant correlations with the CBI subscale emotional burden.

## Discussion

### Group differences

HD gene-carriers exhibited lower scores than their close others for QoL, some self-reported social cognition scales (i.e. IRI perspective taking; emotion contagion), and emotional awareness in relation to interoception, but higher scores for social anxiety and avoidance. This may be the first study to identify these latter associations with HD, although anxiety is known to be common in gene-carriers [22], as are difficulties with social cognition [18]. The current sample included some HD gene-carriers who didn’t have more obvious or observable symptoms, but perhaps those individuals were self-monitoring for the appearance of signs or symptoms that could suggest progression of HD, and this could contribute to social anxiety. In turn, avoidance of social situations may develop not just because of concern about the observations and judgments of others, but to reduce the psychological impact of increased self-monitoring, and/or the need for self-regulation, in social contexts.

The group differences in interoception in the form of emotional awareness help to explain atypical emotional reactivity to evocative stimuli in HD [72], and reports of alexithymia in some samples [24, 35], as well as having implications for social cognition. However, there weren’t group differences for the self-report measures related to empathic concern and mind reading motivation. One possible reason for this is that while patients can have difficulties interpreting mental states and social situations, they may remain motivated to attempt to attend to these. Alternatively, lack of differences on some measures could reflect the range of disease severity included, or the limitations associated with self-report.

The finding of lower QoL in gene carriers would be expected based on previous research, and the lack of an effect for emotional wellbeing in particular may reflect lower scores for that aspect in close others of those with HD as opposed to the wider population. Indeed, scores for the latter subscale, as well as social functioning, pain, and general health appear somewhat lower in the current close other sample than norms reported for the general population for participants of a similar age or without mental health diagnoses [73]. Future social cognitive research in HD should consider the level of deficit on more objective measures and in detail, especially given the discrepancy between emotion contagion and empathic concern highlighted here, as well as the relationships between elevated social anxiety, other more generalised anxiety symptoms and social withdrawal.

### Interoception and affective empathy

The second hypothesis was also partially supported. For non-gene carrying close others, both self-reported emotion contagion and empathic concern were predicted by interoception in terms of the ability to self-regulate responsivity to interoceptive signals, whereas a different pattern was apparent for gene-carriers. First, interoception was not correlated with emotion contagion in patients. Second, while patients’ empathic concern for others was associated with interoception, this was in terms of their ability to regulate attention towards their own inner states. These data could imply that problems with interoceptive signals may impair affective processes, and in turn, aspects of affective empathy in HD, helping to explain social cognitive deficits in many previous studies. This may include patients’ selective deficits in labelling emotion versus age from facial expressions, which covary with differential activity in anterior insula and anterior cingulate [36], brain regions associated with interoception and emotional awareness [74]. More specifically, some of the internal signals experienced by individuals with HD could be interfering with or distracting from other signals that are useful to be aware of during affect recognition and regulation. Such distraction could occur due to excessive or aberrant signals resulting from the disease process and/or difficulties with filtering processes. Interoceptive dysfunction in HD is in accordance with other studies revealing sensory disturbances and evidence of morphometric alterations to the insula and anterior cingulate [75, 76] but could also implicate other areas such as somatosensory cortex and subcortical structures including the thalamus [77].

### Interoception, affective empathy and behavioural difficulties

In HD, low interoception: “not worrying” scores were predictive of higher personal distress (PD; also in the close other group), as well as higher social anxiety and social avoidance. Previous studies indicate that increased awareness of interoceptive signals can contribute to social anxiety disorder [78]. More specifically, high PD predicts high social anxiety in non-clinical samples (healthy students), suggesting that regulated attention towards interoceptive signals plus low levels of associated anxiety seem to be needed for an effective empathic response towards others, while maintaining self-other distinction and mental health [26]. One explanation for the current findings in HD is difficulty in making sense of interoceptive signals in terms of understanding whether the associated affect originated internally or arose through affective resonance with another person (i.e. an external source). Such difficulties would encourage high PD in interpersonal situations and make one’s internal state feel less predictable, thereby increasing anxiety, and potentially leading to a vicious circle of confusion, distress and social anxiety [26].

On the other hand, apathy was not predicted by interoception in the current study. A lack of association could also be due to the AES being rated by close others. However, patients’ self-rated empathic concern and emotion contagion did predict apathy ratings given by close others, such that lower empathic concern predicted higher apathy. These findings support the likelihood that patients can be aware of some of their affective limitations when prompted, and that close others may be aware (consciously or unconsciously) of the same, and that related observations contribute to their perceptions about the patient’s motivational state. In sum, hypothesis 3 was also partly supported, and the emerging pattern seems to suggest that interoceptive dysfunction in HD and related affective changes are likely to contribute to both deficits in affective empathy and motivational behaviours related to social interaction. However, given that apathy can be broken down into components related to affect versus cognition [79] and the measure of apathy used was rather broad and not focussed on affective aspects, future studies exploring interoception in HD should incorporate measures of affective apathy in particular.

### Relationships with gene-carrier functional capacity and QoL

The fourth hypothesis held that patients’ interoception, self-reported social cognition and behavioural difficulties would predict functional capacity and QoL. Attention regulation to interoceptive signals was identified as the sole significant predictor of functional capacity. While it is important to note that this was also the significant predictor of patients’ empathic concern, it is perhaps more likely that this interoceptive ability could reflect a more generalised product of the disease process underlying numerous symptoms, including those that impact physical function and cognition e.g. the ability to focus. In contrast, patients QoL scores on various dimensional subscales were usually predicted by a combination of interoception, PD and social anxiety. Social anxiety was most consistently a predictor for the disease specific scale, whereas interoception and PD predicted more scores on the generic measure. Taken together, the findings are that while both interoception and social anxiety predict QoL in HD gene-carriers, interoceptive dysfunction may exert additional indirect effects via affective empathy and motivational factors, which in turn, influence social behaviour.

### Relationships with close other QoL and carer burden

Our final expectation was that close other QoL and carer burden would be predicted by a combination of patients’ apathy, social anxiety, interoception and social cognition. The prediction in relation to QoL was not supported, as there were no significant correlations between close other scores and gene-carrier measures of apathy, social anxiety and avoidance, interoception or social cognition. There were also no significant correlations between QoL scores for close others and any of the subscale scores for patients’ QoL. This was most surprising for apathy, given relationships identified in previous research [7, 80]. Possibly, the lack of association could reflect the sensitivity of the QoL measure, or discrepancies between studies in relation to the impairment/disease severity of the gene-carrier group. Either way, some findings were in line with our expectations, in the case of carer burden. Overall carer burden, and developmental burden specifically, were predicted by high apathy, and by interoception: not distracting scores (i.e. by the gene-carrier having trouble distracting themselves from internal signals). High physical burden was predicted by low emotional awareness in patients, while high social burden was predicted by high apathy and low interoception: listening. High interoception; attention regulation scores predicted lower time-dependent burden for carers. This implies that the reactivity of HD gene-carriers to interoceptive signals in terms of listening, attending, and emotional awareness seems to significantly influence the burden felt by their carers. The data support the possibility that the way this burden manifests may morph from an emotional difficulty as expressed in the patient, to a physical burden as experienced by carers, in turn raising the potential for somatisation, and perhaps helping to explain the relatively high scores for the QoL pain subscale in the close other group.

### Limitations

In looking at the MAIA-2 subscales more closely, it was those involved in the perception and regulation of physiological bodily signals [38] i.e. “listening”; “noticing”; “not distracting”; “attention regulation” and “self regulation”, that were most often predictive of patients’ empathy, functional capacity and QoL. However, it was awareness and interpretation (i.e. “emotional awareness”) that was significantly more challenging for those with HD when compared to close others, and which may also underlie the correlation between the “not worrying” subscale with gene-carriers’ social functioning (QoL). While some of the latter relationships implicate problems at the level of cognitive reflection, this is perhaps a step beyond what can be inferred from the measure. Indeed, it is possible that some of the identified relationships are influenced by unknown mediating variables, such as introspection, metacognition, or other variables relevant to self-awareness*.* The capacity to regulate and focus attention on competing physiological sensations should be further explored in HD, as while significant group differences were not apparent in the current study, these skills did correlate with functional capacity and QoL, and it may have been hard to capture, or for patients to accurately self-report, all of the problems they have with perceiving and interpreting bodily signals on the single scale that was administered.

The current study was limited by the lack of more objective measures of social cognition, and subject to the potential reliability issues associated with self-report. Therefore, inferences drawn about the nature and clinical significance of the findings remain somewhat speculative. While participants of a range of disease stages were included, those further along in the disease process may be less well represented. Many of the relationships revealed by this study need to be followed up in future research, especially to determine directional effects. While including a range of social, emotional, clinical and behavioural measures in this exploratory study may be considered a strength, it is challenging to interpret some findings. Other factors that weren’t explored, such as disease burden, executive function or social and environmental variables, could mediate the identified relationships. In relation to apathy specifically, the multidimensional nature of apathy in HD can include cognitive/executive, initiation/activation and emotional components [81, 82]. Therefore while this study has focussed on relationships with self-reported social cognition and identified relationships with empathic concern, there could still be a role for cognition in shaping behavioural problems. This includes social anxiety, which could be high in different patients for different reasons e.g. cognitive impairment versus awareness of motor symptoms versus emotional sensitivity etc. There are also particular complications when interpreting social avoidance behaviours, as these could reflect not just social anxiety, but also apathy, fatigue, the impact of motor or cognitive dysfunction, or strategies to manage self-monitoring. Indeed, it can be hard to know whether many behavioural features pertain directly to HD related neuropathology, more general psychological factors or disease related social factors. Hence care is required when interpreting the data with consideration given to the challenges in ascertaining whether behavioural difficulties constitute symptoms of HD [83].

## Conclusions and potential clinical implications

In conclusion, HD is characterised by interoceptive difficulties which impair patients’ emotional awareness and impact empathic concern, functional capacity and QoL via poor regulation of attention to internal physiological states. Future research may substantiate that one side of poor regulation of attention to internal signals is high personal distress and social anxiety, while the other is low affective empathy and perceived apathy. Imbalances in either direction could encourage social withdrawal, and when gene-carriers struggle to maintain interoceptive equilibrium, the current data imply this is likely to increase the burden experienced by their care givers. Indeed, the affective deficits associated with interoceptive dysfunction can influence close others’ evaluation of patients’ behavioural problems, as shown by the relationship between carer ratings of apathy and patients’ self ratings of empathic concern. These findings highlight the likely role of interoceptive dysfunction in the experiences of gene-carriers and their close others who sense a change in the person with HD that they find hard to explain. Interoceptive processes occur below the level of consciousness and are interconnected with motivations, impulses, cognition, emotions and adaptive reactions [28]. Difficulties with interoception and related automatic affective processes may remain largely hidden, perhaps initially only exerting subtle effects on the non-verbal resonance or inter-personal synchrony that previously characterised gene-carriers’ close relationships. With disease progression, close others may find it easier to pin down overt examples of incongruent affect or reduced rapport as these fuel a growing sense of disconnection. Therapeutic options for HD remain limited, especially those to address psychological problems [84]. Future research should continue to explore the identified relationships while seeking to improve measurement of patients’ social and emotional symptoms in order to provide a framework for new interventions and ultimately enhance the support and healthcare options available to individuals affected by HD.

## Supplementary Information

Below is the link to the electronic supplementary material.

Supplementary file1 (PDF 137 KB)

## Data Availability

The datasets used during the current study are available from the corresponding author on reasonable request.
