# Supplementary material for: Interoception predicts empathy, social anxiety and quality of life in Huntington’s disease
Source: J Neurol. 2026 Nov 12;273(12):713. doi: 10.1007/s00415-026-14248-4 (PMC13759550; doi:10.1007/s00415-026-14248-4)
Supplement: Supplementary file 1 [file 415_2026_14248_MOESM1_ESM.pdf]

Journal of Neurology
Interoception predicts empathy, social anxiety and quality of life in Huntington's disease
Clare M. Eddy[1*] & Hugh Rickards[1]
College of Medicine and Health, University of Birmingham and BSMHFT National Centre for Mental Health, Birmingham, UK
*Corresponding author   Email: c.eddy@bham.ac.uk; clare.eddy1@nhs.net

Supplementary Table 1. Correlational analysis

Hypotheses and measures	DV	Correlated IVs	Statistics
**2a. Interoceptive skills will predict affective empathy (patients)**<br>Variables: Empathic concern, ECS (total), MAIA total and 8 subscales	Empathic concern	Attention regulation	Sr=.465, p=.006
		Self regulation	r=.378, p=.028
		MAIA trusting	r=.373, p=.030
	Personal distress	MAIA not worrying	r=-.575, p=<.001
**…and motivational symptoms (patients)**	LSAS anxiety	MAIA not worrying	r=.-496, p=.003
	LSAS avoidance	MAIA not worrrying	Sr=-.487, p=.004
	AES	MAIA attention regulation	Sr=.380, p=.032
**2b. Interoceptive skills will predict affective empathy (close others)**<br>Variables: Empathic concern, ECS total, MAIA (total and 8 subscales)	Empathic concern	MAIA self regulation	r=.388, p=.026
	Personal distress	MAIA not worrying	r=-.356, p=.042
	ECS total	MAIA self regulation	r=.358, p=.041
**3. Social skills will predict patient apathy and social anxiety**<br>Variables: AES, IRI (4 subscales), ECS (total), MRM, LSAS (2 subscales)	AES	Fantasy	r=.370, p=.037
		Perspective taking	r=.365, p=.040
		Empathic concern	r=.577, p<.001
		ECS	r=.558, p<.001
	LSAS anxiety	Personal distress	r=.566, p<.001
	LSAS avoidance	Personal distress	Sr=.419, p=.014
**4. Social anxiety, apathy, social skills and interoception will predict patients' functional capacity and QoL**<br>Variables: Total functional capacity score, LSAS (2 subscales), SF36 (8 subscales), HDQoL (total and 5 subscales), MAIA (8 subscales), ECS (total), MRM, IRI (4 subscales)	Total functional capacity	Empathic concern	r=.424, p=.019
		MAIA attention regulation	Sr=.563, p=.001
		MAIA self regulation	Sr.409, p=.025
	SF36 Physical functioning	LSAS anxiety	Sr=-.457, p=.007
		LSAS avoidance	Sr=-.385, p=.025
		MAIA not worrying	Sr=.607, p<.001
		Personal distress	Sr=-.524, p=.001
	SF36 Role physical	LSAS anxiety	Sr=-.418, p=.014
		MAIA not worrying	Sr=.470, p=.005
		MAIA listening	Sr=-.468, p=.005
		Personal distress	Sr=-.542, p<.001
	SF36 Role emotional	LSAS anxiety	Sr=-.546, p<.001
		LSAS avoidance	Sr=-.496, p=.003
		MAIA not worrying	Sr=.420, p=.014
		MAIA listening	Sr=-.447, p=.008
		Personal distress	Sr=-.367, p=.033
	SF36 Energy	LSAS anxiety	r=-.464, p=.006

		LSAS avoidance	Sr=.493, p=.003
		MAIA not worrying	r=.458, p=.006
	SF36 Emotional wellbeing	LSAS anxiety	r=-.617, p<.001
		LSAS avoidance	Sr=-.576, p<.001
		MAIA not worrying	r=.533, p=.001
		MAIA listening	Sr=-.455, p=.007
		Personal distress	r=-.440, p=.009
	SF36 Social functioning	LSAS anxiety	Sr=-.430, p=.011
		LSAS avoidance	Sr=-.378, p=.027
		MAIA not worrying	Sr=.486, p=.004
		MAIA listening	Sr=-.358, p=.037
		Personal distress	Sr=-.344, p=.046
	SF36 Pain	MAIA not worrying	Sr=.355, p=.039
		Personal distress	Sr=-.476, p=.004
	GEN HEALTH	MAIA not worrying	r=.404, p=.018
		Personal distress	r=-.445, p=.008
	HDQoL Total	LSAS anxiety	r=-.725, p<.001
		LSAS avoidance	Sr=-.645, p<.001
		MAIA emotional awareness	r=-.378, p=.028
		MAIA noticing	r=.525, p=.001
		MAIA listening	Sr=-.340, p=.049
		Personal distress	r=-.495, p=.003
	HDQoL physical	LSAS anxiety	Sr=-.645, p<.001
		LSAS avoidance	Sr=-.532, p<.001
		MAIA not worrying	Sr=-.568, p<.001
		Personal distress	Sr=-.564, p<.001
	HDQoL Cognitive	LSAS anxiety	Sr=-.644, p<.001
		LSAS avoidance	Sr=-.608, p<.001
		MAIA not worrying	Sr=.488, p=.003
		Personal distress	Sr=-.490, p=.003
	HDQoL Mood	LSAS anxiety	Sr=-.662, p<.001
		LSAS avoidance	Sr=-.644, p<.001
		MAIA emotional awareness	Sr=-.419, p=.014
		MAIA not worrying	Sr=.474, p=.005
		LIST	Sr=-.378 p=.028
	HDQoL Worry	LSAS anxiety	r=-.596, p<.001
		LSAS avoidance	r=-.533, p=.001
		MAIA emotional awareness	r=-.448, p=.008
		MAIA noticing	Sr=-.472, p=.005
		MAIA not worrying	r=.472, p=.005
		Fantasy	r=-.408, p=.017
		Personal distress	r=-.487, p=.004
	HDQoL Services	LSAS anxiety	Sr=-.447, p=.008
		LSAS avoidance	Sr=-.360, p=.037
		MAIA emotional awareness	Sr=-.431, p=.011
		MAIA not worrying	Sr=-.404, p=.018
**5. Patients' apathy, social anxiety and social skills will**	CBI Total	AES	Sr=-.455, p=.029
		MAIA not distracting	Sr=.417, p=.048

**predict close others' QoL and carer burden**<br>**Variables:** CBI (total and 5 subscales) + AES + MAIA (8 subscales) + ECS (total)<br><br>SF36 (8 subscales) – No significant correlations		MAIA emotional awareness	Sr=-.505, p=.014
	CBI Physical	MAIA emotional awareness	Sr=-.517, p=.004
		MAIA listening	Sr=-.473, p=.023
	CBI Time	MAIA attention regulation	Sr=-.445, p=.033
	CBI Developmental	MAIA not distracting	Sr=-.426, p=.043
		MAIA emotional awareness	Sr=-.448, p=.032
	CBI Social	AES	Sr=-.579, p=.004
		MAIA emotional awareness	Sr=-.474, p=.022
		MAIA listening	Sr=-.426, p=.043

KEY: CBI: Carer Burden Inventory; HDQoL: Huntington's Disease Quality of Life scale; IRI; Interpersonal Reactivity Index; LSAS: Liebowitz Social Anxiety Scale; MAIA: Multidimensional Assessment of Interoceptive Awareness 2; p: p-value; SF36: Rand Health Questionnaire; Sr: Spearman's rank correlation coefficient (otherwise Pearson's r).
